# Double-chambered left ventricle: diagnosis by CMR and review of the literature

**DOI:** 10.1186/s43044-023-00341-w

**Published:** 2023-03-07

**Authors:** Sheema Saadia, Aiysha Nasir, Fateh Ali Tipoo Sultan

**Affiliations:** Section of Cardiology, Department of Medicine, The Aga Khan University Hospital, Karachi, Pakistan

**Keywords:** Congenital heart disease, Double-chambered left ventricle, Cardiac magnetic resonance imaging

## Abstract

**Background:**

A double-chambered left ventricle (DCLV) is an extremely rare congenital malformation. The exact prevalence of DCLV is not known, although studies have reported prevalence of 0.04–0.42%. This abnormality is characterized by the sub-division of left ventricle into two chambers, the main left ventricular chamber (MLVC) and the accessory chamber (AC) by a septum or muscle band.

**Case presentation:**

We are reporting two cases of DCLV, one in an adult male and an infant, who were referred for undergoing cardiac magnetic resonance (CMR) imaging. The adult patient was asymptomatic, whereas the infant had the diagnosis of left ventricular aneurysm on fetal echocardiography. On CMR, we confirmed the diagnosis of DCLV in both patients, as well as moderate aortic insufficiency in the adult patient. Both patients were lost to follow-up.

**Conclusions:**

The double-chambered left ventricle (DCLV) is commonly detected in infancy or childhood. Although echocardiography can help detect double-chambered ventricles, MRI provides a better knowledge of this problem and can also be used to diagnose other related heart disorders.

## Background

Double-chambered left ventricle (DCLV) is an extremely rare congenital heart disease. Only a few cases of DCLV with variable morphologies have been reported in local and international literature [1–3]. It is characterized by two chambers of DCLV are in parallel with superior/ inferior chambers or left/right chambers [4]. It has been more commonly described and reported in right ventricle, although cases have been reported in left ventricle (LV) as well. The incidence of double-chambered right ventricle (RV) is seen in only 0.5–2.0% of all cases of congenital heart disease and incidence of double-chambered LV is even more less [5]. The exact prevalence of congenital DCLV is not known, although studies have reported prevalence of 0.04–0.42% [6, 7].

The pathogenesis or exact etiology of this rare anomaly remains elusive but DCLV is considered to result from hypoplasia of regional myocardial intra-trabecular sinusoids or from intra-myocardial aneurysm that develops during embryogenesis [1]. Another theory is that of incomplete regression of the trabeculations, probably a variant of left ventricular non-compaction [8, 9]. DCLV is characterized by division of LV chamber into two chambers by abnormal muscular tissue or accessary septum which by nature could be muscle band most of the time (55%) or it could be a membranous structure, fibromuscular ridge or prominent trabeculations as illustrated in a systemic review by Yuan [10]

Like other cardiac diseases i.e. hypertrophic cardiomyopathy, dilated cardiomyopathy, left ventricular non-compaction and Ebstein anomaly there must be genetic cause of this rare anomaly. Wang et al. [11] identified in a study, a heterozygous missense rare variant in MYH7, resulting in an amino acid change from aspartic acid to valine at position 333, in a DCLV pedigree.

Literature review reveals that five overlapping / interchangeable and poorly defined terms are used in reporting this rare entity: LV aneurysm, LV diverticulum, Cysts, DCLV, and accessory left ventricle, which confounds the prevalence reported. The two most important differential diagnoses of DCLV are LV aneurysm and diverticulum. True LV aneurysm is a saccular protrusion of the LV wall due to mechanical weakness containing all the three cardiac tissue layers, with scarred/fibrotic wall having wide based neck with the LV and is either akinetic or dyskinetic [1]. Pseudoaneurysm does not contain all the three layers of cardiac tissue, have a narrow neck, and may exhibit paradoxical (dyskinetic) movement during systole. DCLV and LV diverticulum contain all layers of cardiac tissue that typically contract synchronously with the rest of the ventricle [1, 12]. Diverticulum, however, differs from DCLV in having a narrow neck connecting the diverticulum to the left ventricle [13, 14]. DCLV is generally suspected when an abnormally configured left ventricle with two distinct contracting chambers is seen by echocardiography [1, 13], but the accessory chamber is not necessarily contractile in all patients with this condition and can also exhibit akinetic to dyskinetic contractility in some patients as illustrated in a case report and systemic review by Yuan Shi-Min and sharma et al. [10, 15].

This condition can be diagnosed by Cardiac CT or echocardiography, even fetal echo can sometimes give a clue to the diagnosis of DCLV; however, it can be misdiagnosed as atrial or ventricular septal defect. CMR is a better modality to confirm the diagnosis because of its better spatial resolution and tissue characterization ability specially when differentiating normal myocardium from fibrosis [16]. Generally these patients remain asymptomatic and are diagnosed incidentally during evaluation for other conditions and have a benign prognosis [2, 12]. There are only a few case reports of complications including one case of coronary embolism and 3 cases of ventricular arrhythmias associated with DCLV, including a 22-year-old man who presented with non-sustained ventricular tachycardia and 49 year-old man who presented with monomorphic VT [15, 17] and a 68-year-old man who was admitted with ventricular fibrillation and found to have DCLV [18].

There is no extensive data on treatment options and on outcomes, due to rarity of this condition. Nonetheless, few studies [10, 19] have demonstrated that treatment and follow-up have to be individualized to each patient based on their clinical presentation, concomitant cardiac structural/congenital abnormalities, and possible complications like anticoagulation for systemic embolism, antiarrhythmic drugs with or without implantation of an implantable cardioverter defibrillator for symptomatic ventricular arrythmias, surgical resection especially in symptomatic patients with left ventricular obstruction or a conservative management (observation) with a follow-up for asymptomatic patients. Indications for surgical resection include intra-left ventricular pressure gradient of 110 mmHg [19], a high flow velocity of > 2 m/s [20], and severe left ventricular outflow obstruction [21]. Surgical excision of the accessory chamber with interposition patch reconstruction and cardiac transplant are two appropriate options [22]. Follow-up of such patients is necessary to assess for the development of symptoms, repeating an imaging modality to look for left ventricular obstruction, left ventricular function, intracardiac volumes as well as progression of other related structural or valvular abnormalities. These few studies on the outcomes of DCLV patients have also yielded positive findings and a favorable prognosis following both conservative and surgical therapies [10, 14, 19, 21]

## Case presentation

A 35-year-old man with history of cardiac surgery during childhood for which no record was available, was referred to our institute for cardiac magnetic resonance (CMR) imaging after the echocardiographic evaluation that was also done outside of our hospital and no images were available for the review.

**Fig. 1:**
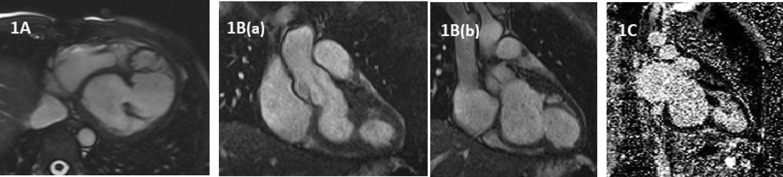
**A**: CMR Axial view showing double-chambered left ventricle. **B**: CMR LVOT view showing double-chambered LV in systole (a) and diastole (b). **C**: Late gadolinium image showing double-chambered LV with no hyperenhancement

CMR was done on 1.5 Tesla, Siemens Avanto scanner. There were prominent muscle bundles in the left ventricle, dividing the left ventricle into two chambers (double-chambered left ventricle) (Fig. 1A–B). Left ventricular volumes were in the upper range of normal with normal left ventricular systolic function/ejection fraction (LVEF 59%). Overall left ventricular mass was normal. Aortic valve was tri-commissural with moderate aortic regurgitation with regurgitation fraction of 34%. A small thin sub-aortic membrane was also visualized. Right ventricular volumes were normal with normal right ventricular systolic function. There was no evidence of myocardial enhancement in the left or right ventricle, on late gadolinium images. (Fig. 1C). Patient was managed conservatively with a plan for follow-up imaging, as he was asymptomatic with moderate aortic regurgitation and no clinical signs of cardiac decompensation or heart failure. Follow-up imaging was advised to look for the severity of aortic regurgitation, left ventricular volumes and function, but the patient did not come back on follow-up, and we were unable to record his clinical progress on the conservative management.

A 26 days old baby girl with the diagnosis of large left ventricular out-pouching/aneurysm (14 × 10 mm) on fetal echocardiography that was done outside of our institute and no record of images were available except a written report with suspicion of DCLV versus true aneurysm, was referred to us for CMR.

**Fig. 2:**
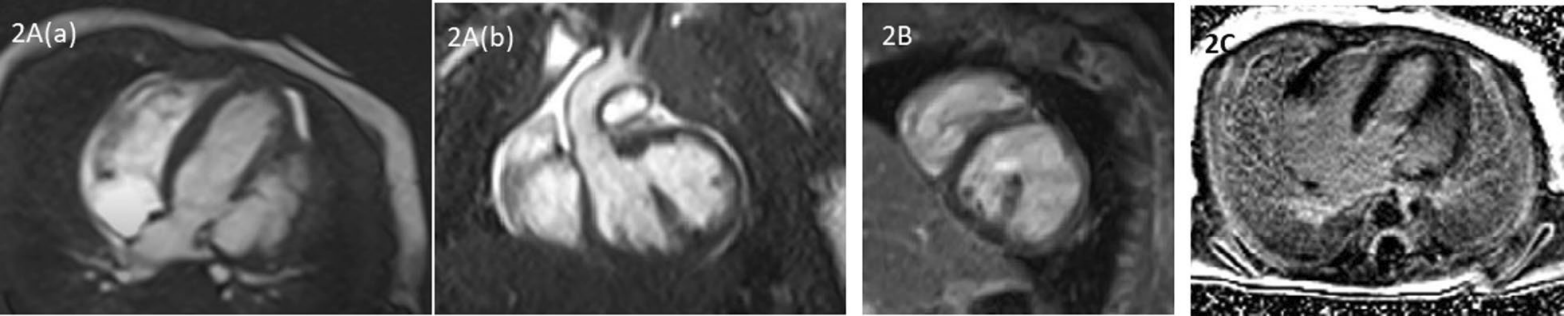
**A**: Apical 4 chamber (a) and LVOT (b) view showing double-chambered left ventricle. **B**: Early gadolinium image showing double-chambered LV with no evidence of thrombus. **C**: Late gadolinium image with no evidence of myocardial enhancement

Postal natal echocardiography showed large out-pouching, most probably aneurysm versus diverticulum of left ventricular posterior wall with wide open connection with left ventricle and some dyskinetic contractility. The infant was electively intubated for CMR, which was done on 1.5 Tesla, Siemens Avanto scanner. CMR showed a large out-pouching of left ventricle from basal lateral wall, with a broad base and measuring up to 27 mm in the long axis along lateral wall and 14 mm in the short axis. It was giving the appearance of double-chambered left ventricle; with contracting but hypokinetic walls (Fig. 2A). Its walls were of normal thickness except in certain regions and was composed of all the three layers. Overall left ventricular systolic function was moderately reduced (LVEF 33%). There was no evidence of thrombus on early gadolinium images. (Fig. 2B) No myocardial enhancement was observed on late gadolinium images. (Fig. 2C). This patient was likewise lost to follow-up.

Both our patients did not undergo transesophageal echocardiographic assessment and/or cardiac catheterization.

## Conclusions

Double-chambered left ventricle (DCLV), a rare cardiac malformation is characterized by the sub-division of the left ventricle and is usually diagnosed in the neonatal or pediatric age but rarely it remains asymptomatic and undiagnosed till adulthood as in one of our cases. Even though echocardiography or fetal echocardiography, as in one of our cases, can aid in the detection of double-chambered ventricles, however, MRI allows a better delineation and understanding of this condition because of its higher spatial resolution and tissue characterization ability. CMR is also helpful in the assessment of other associated cardiac abnormalities including congenital, structural, or valvular heart diseases. Due to the rarity of this illness, there is little evidence on treatment approaches and the prognosis.

## Data Availability

Not applicable.

## Abbreviations

DCLV: Double-chambered left ventricle
MLVC: Main left ventricular chamber
AC: Accessory chamber
CMR: Magnetic resonance
LV: Left ventricle
RV: Right ventricle
MYH7: Myosin heavy chain 7
CT: Computerized tomography
VT: Ventricular tachycardia
LVEF: Left ventricular ejection fraction
