# Correction: Double-chambered left ventricle: diagnosis by CMR and review of the literature

**DOI:** 10.1186/s43044-023-00348-3

**Published:** 2023-03-23

**Authors:** Sheema Saadia, Aiysha Nasir, Fateh Ali Tipoo Sultan

**Affiliations:** Section of Cardiology, Department of Medicine, The Aga Khan University Hospital, Karachi, Pakistan

**Correction: The Egyptian Heart Journal (2023) 75:15** 10.1186/s43044-023-00341-w

Following publication of the original article [1], it was noticed that the *Consent for publication* statement was incorrect. The correct statement is as follows:

## Consent for publication

Written informed consent was obtained from the first adult patient and from the parents of the second patient (infant) on a pre-defined consent paper.
